# Cardiomiopatia PRKAG2: Um Estudo Caso-Controle sobre o Rendimento Diagnóstico da Histopatologia e da Análise Ultraestrutural da Biópsia Endomiocárdica

**DOI:** 10.36660/abc.20240616

**Published:** 2026-02-27

**Authors:** Kinulpe Honorato-Sampaio, Carla de Oliveira, Stanley de Almeida Araújo, Frederico Soares Correa, Glauber Monteiro Dias, Antônio Fernandino de Castro Bahia, Vinícius Lopes Cantuária, Arash Yavari, Maria da Consolação Vieira Moreira, José Luiz Barros Pena, Geraldo Brasileiro, Eduardo Back Sternick

**Affiliations:** 1 Faculdade de Medicina Universidade Federal do Vale do Jequitinhonha e Mucuri Diamantina MG Brasil Faculdade de Medicina, Universidade Federal do Vale do Jequitinhonha e Mucuri, Diamantina, MG – Brasil; 2 Faculdade de Ciências Médicas Belo Horizonte MG Brasil Faculdade de Ciências Médicas, Belo Horizonte, MG – Brasil; 3 Hospital das Clínicas Universidade Federal de Minas Gerais Belo Horizonte MG Brasil Hospital das Clínicas - Universidade Federal de Minas Gerais, Belo Horizonte, MG – Brasil; 4 Hospital Biocor Rede D’Or São Luiz Nova Lima MG Brasil Hospital Biocor, Rede D’Or São Luiz, Nova Lima, MG – Brasil; 5 Laboratório de Biologia Celular e Tecidual Centro de Biociências e Biotecnologia Universidade Estadual do Norte Fluminense Darcy Ribeiro Campos dos Goytacazes RJ Brasil Laboratório de Biologia Celular e Tecidual, Centro de Biociências e Biotecnologia - Universidade Estadual do Norte Fluminense Darcy Ribeiro, Campos dos Goytacazes, RJ – Brasil; 6 Departamento de Cardiologia Hospital Nossa Senhora das Graças Sete Lagoas MG Brasil Departamento de Cardiologia - Hospital Nossa Senhora das Graças, Sete Lagoas, MG – Brasil; 7 Experimental Therapeutics and Division of Cardiovascular Medicine Radcliffe Department of Medicine University of Oxford Oxford United Kingdom Experimental Therapeutics and Division of Cardiovascular Medicine, Radcliffe Department of Medicine, University of Oxford, Oxford – United Kingdom; 8 Departamento de Clínica Médica Faculdade de Medicina Universidade Federal de Minas Gerais Belo Horizonte MG Brasil Departamento de Clínica Médica - Faculdade de Medicina, Universidade Federal de Minas Gerais, Belo Horizonte, MG – Brasil; 9 Departamento de Anatomia Patológica Faculdade de Medicina Universidade Federal de Minas Gerais Belo Horizonte MG Brasil Departamento de Anatomia Patológica, Faculdade de Medicina - Universidade Federal de Minas Gerais, Belo Horizonte, MG – Brasil; 10 Hospital Felício Rocho Belo Horizonte MG Brasil Hospital Felício Rocho, Belo Horizonte, MG – Brasil

**Keywords:** Proteínas Quinases Ativadas por AMP, Cardiomegalia, Microscopia Eletrônica de Transmissão

## Abstract

**Fundamento:**

As características histopatológicas da cardiomiopatia *PRKAG2* foram relatadas de forma fragmentada.

**Objetivo:**

Nosso objetivo foi avaliar sistematicamente as características patológicas cardíacas da cardiomiopatia *PRKAG2* em uma grande coorte de pacientes e avaliar seu potencial diagnóstico em comparação com o sequenciamento genético.

**Métodos:**

Realizamos um estudo observacional, transversal, caso-controle, incluindo 18 pacientes com cardiomiopatia associada ao gene *PRKAG2* e 11 receptores de transplante cardíaco como grupo controle. Todos os pacientes foram submetidos a biópsia endomiocárdica percutânea do ventrículo direito. As amostras de tecido foram analisadas por meio de coloração com hematoxilina-eosina (H&E), coloração com ácido periódico-Schiff para glicogênio, tricrômico de Masson para fibrose e avaliação ultraestrutural por microscopia eletrônica de transmissão. A significância estatística foi definida em p < 0,05 para todas as análises.

**Resultados:**

Os corações com cardiomiopatia associada ao gene *PRKAG2* apresentaram aumento significativo dos cardiomiócitos, mitocôndrias com aparência normal, extensa vacuolização da maioria das miofibras, fibrose intersticial mínima (apenas dois pacientes apresentaram fibrose leve) e ausência de infiltração de células inflamatórias. A microscopia eletrônica de transmissão revelou abundante glicogênio citosólico, principalmente na região perinuclear, com depósitos adicionais nas áreas intermiofibrilares e subsarcolemáticas. Esse acúmulo pronunciado de glicogênio, observado consistentemente em todos os pacientes com *PRKAG2* , estava ausente nos controles.

**Conclusão:**

O exame histológico e ultraestrutural de amostras de biópsia endomiocárdica do ventrículo direito revela um conjunto distinto de características que sugerem fortemente a cardiomiopatia associada ao gene *PRKAG2* .

## Graphical abstract

**Figura Central:**
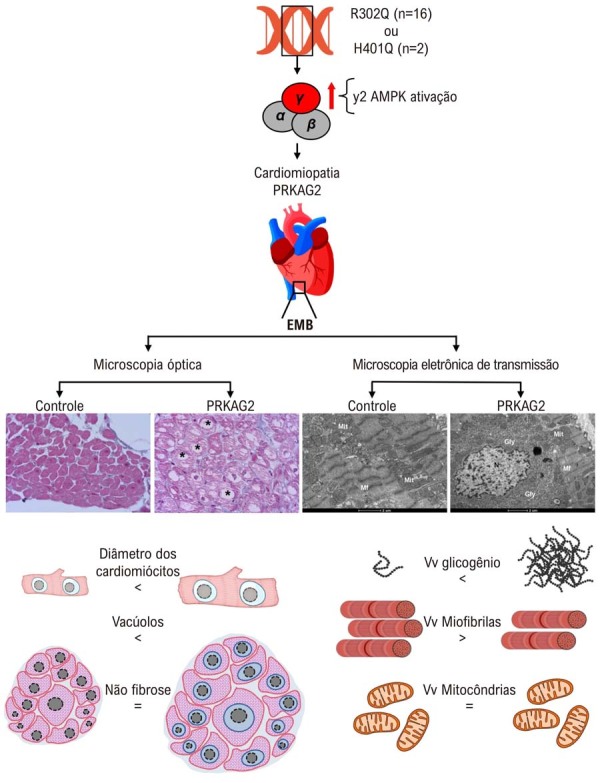
Cardiomiopatia PRKAG2: Um Estudo Caso-Controle sobre o Rendimento Diagnóstico da Histopatologia e da Análise Ultraestrutural da Biópsia Endomiocárdica

A análise histológica e ultraestrutural da biópsia endomiocárdica do ventrículo direito revela um conjunto consistente de características sugestivas de cardiomiopatia PRKAG2, incluindo aumento do tamanho dos cardiomiócitos, vacuolização pronunciada, ausência de fibrose intersticial, acúmulo substancial de glicogênio e densidade volumétrica mitocondrial preservada.

## Introdução

Mutações no gene *PRKAG2* , que codifica a subunidade regulatória γ2 da proteína quinase ativada por AMP, levam a um fenótipo altamente penetrante de hipertrofia ventricular, pré-excitação, taquiarritmias atriais, bradicardia sinusal progressiva e anormalidades da condução cardíaca.^1^ A principal característica patológica – acúmulo anormal de glicogênio nos cardiomiócitos – levou inicialmente à sua classificação como uma cardiomiopatia de armazenamento de glicogênio e uma fenocópia da cardiomiopatia hipertrófica sarcomérica.^2^

Desde suas primeiras descrições, as características morfológicas da cardiomiopatia *PRKAG2* têm sido relatadas principalmente utilizando amostras de biópsia endomiocárdica (BEM) do septo ventricular direito,^2 - 15^ um número limitado de autópsias de pacientes que morreram subitamente,^2 , 14 , 16 , 17 , 18^e corações explantados de receptores de transplante.^7 , 19^ Os achados patológicos parecem depender do momento da biópsia dentro da história natural da doença, com anormalidades mais pronunciadas observadas em pacientes com manifestações clínicas graves.^1 - 19^Por exemplo, corações explantados de receptores de transplante com insuficiência cardíaca terminal frequentemente exibem características distintas em comparação com aqueles de pacientes jovens que sucumbiram à morte súbita cardíaca.

Desde a identificação do gene *PRKAG2,* ^20 , 21^ o sequenciamento genético se tornou o padrão ouro para o diagnóstico. No entanto, o papel potencial da avaliação da patologia cardíaca nunca foi examinado sistematicamente. A BEM do ventrículo direito (VD) é uma ferramenta diagnóstica segura que foi amplamente utilizada na avaliação de suspeitas de cardiomiopatias por armazenamento de glicogênio antes da ampla disponibilidade dos testes genéticos.^22 , 23^ No Brasil e em outros países de baixa renda, o acesso aos testes genéticos permanece limitado, enquanto a BEM é coberta pelo sistema público de saúde. Não está claro, porém, se a BEM oferece acurácia diagnóstica comparável à do sequenciamento genético, que apresenta um rendimento relatado de 100%.

Assim, realizamos um estudo transversal caso-controle para avaliar sistematicamente as características histológicas e ultraestruturais da cardiomiopatia associada ao gene *PRKAG2* , utilizando: (1) uma coorte de 18 pacientes de cinco famílias portadoras das variantes *R302Q* (a variante patogênica mais frequentemente relatada) e *H401Q* do gene *PRKAG2* ; (2) um grupo controle de 11 corações transplantados estruturalmente normais. A análise histológica e ultraestrutural da BEM do VD revelou um conjunto de características sugestivas de cardiomiopatia associada ao gene *PRKAG2* , incluindo aumento do tamanho dos cardiomiócitos, vacuolização pronunciada, ausência de fibrose intersticial, acúmulo substancial de glicogênio e densidade volumétrica mitocondrial preservada (Figura Central).

## Métodos

### Desenho do estudo

Este estudo observacional prospectivo de caso-controle foi aprovado pelo Comitê de Ética em Pesquisa da Universidade Federal de Minas Gerais (SISNEP CAAE: 0511.0.203.418-11). O consentimento livre e esclarecido para a realização da BEM foi obtido de todos os participantes. O estudo foi delineado para avaliar o desempenho diagnóstico da BEM em pacientes com cardiomiopatia por *PRKAG2* geneticamente confirmada, utilizando receptores de transplante como grupo controle.

### População do estudo

O grupo de casos compreendeu 18 pacientes com variantes patogênicas do gene *PRKAG2* (16 com a mutação *R302Q* e dois com a mutação *H401Q* , recentemente relatada pelo nosso grupo^12^). O grupo controle consistiu em 11 receptores de transplante cardíaco sem evidência clínica de rejeição do enxerto, submetidos a BEM de rotina em até 10 dias após o transplante para monitoramento de rejeição. Os corações transplantados apresentavam estrutura e eletrocardiografia normais, conforme avaliado por ecocardiografia e eletrocardiografia.

### Análise genética

O DNA genômico de pacientes com cardiomiopatia *PRKAG2* , isolado de leucócitos do sangue total, foi sequenciado utilizando um painel de 52 genes para cardiomiopatia. Uma biblioteca genômica foi preparada com um painel personalizado AmpliSeq e sequenciada utilizando um sistema PGM Ion Torrent. As sequências foram alinhadas ao genoma de referência hg19 e as variantes de nucleotídeos foram filtradas seguindo as diretrizes do *American College of Medical Genetics and Genomics* e da *Association for Molecular Pathology* .^24 , 25^ Os membros da família foram submetidos a triagem específica para mutações por sequenciamento de Sanger utilizando um analisador genético ABI 3500XL (Thermo Fisher Scientific, Waltham, MA, EUA).

### Biópsia endomiocárdica

Sob anestesia local, um operador experiente realizou BEM utilizando uma pinça de biópsia miocárdica Cook^®^ (Bloomington, IN, EUA), visando o septo interventricular direito para obter cinco amostras de tecido. Três fragmentos foram fixados em formalina a 10% por 12 horas à temperatura ambiente para microscopia óptica. Os dois restantes foram fixados em solução de Karnovsky (2,5% de glutaraldeído e 2% de paraformaldeído em tampão cacodilato 0,1 M, pH 7,4) durante a noite a 4 °C para microscopia eletrônica. Nenhum paciente apresentou complicações.

### Microscopia ótica

Fragmentos de BEM fixados em formalina foram incluídos em parafina, seccionados (2 µm) e corados com hematoxilina-eosina (H&E), ácido periódico-Schiff (PAS) e tricrômico de Masson. O diâmetro dos cardiomiócitos (≥12 miócitos por paciente) foi medido utilizando o software ImageJ v1.49 (National Institutes of Health, EUA). As fotomicrografias foram obtidas com aumento de 400× utilizando um microscópio Axio Imager Z2 - Apotome 2 (Zeiss, EUA).

### Microscopia eletrônica de transmissão

As amostras foram pós-fixadas em OsO_4_ a 1%, coradas durante a noite com acetato de uranila a 2%, desidratadas em etanol e incluídas em Epon 812 (EMS, EUA). Cortes ultrafinos (50 nm) foram obtidos utilizando um ultramicrótomo com lâmina de diamante, montados em grades de cobre e corados com citrato de chumbo de Reynolds. A microscopia eletrônica de transmissão (MET) foi realizada a 80 kV utilizando um microscópio FEI Tecnai G2-12 Spirit, equipado com uma câmera CCD SIS-MegaView 3. As micrografias foram obtidas com ampliação de 9.900×. Imagens foram selecionadas aleatoriamente de regiões centrais de cardiomiócitos e analisadas utilizando o software ImageJ. As densidades volumétricas (Vv) de glicogênio, mitocôndrias e miofibrilas foram determinadas usando o método clássico de contagem de pontos com uma grade de 165 pontos (500 × 500 nm) projetada em cada imagem, seguindo Cantuária et al.^26^ Pelo menos 10 cardiomiócitos por paciente foram submetidos à análise morfométrica.

### Análise estatística

Como a cardiomiopatia *PRKAG2* é uma doença extremamente rara, a amostra do estudo foi definida por conveniência, incluindo todos os pacientes elegíveis disponíveis durante o período do estudo. As imagens de microscopia foram analisadas de forma cega por dois investigadores. Quaisquer discrepâncias foram resolvidas por consenso com um terceiro investigador. Os dados foram analisados utilizando o GraphPad Prism v6.1 (GraphPad Software, EUA) e apresentados como média ± desvio padrão (DP). As variáveis categóricas foram apresentadas como frequências absolutas e relativas. Os dados morfométricos apresentaram distribuição normal (teste de Shapiro-Wilk). A correlação de Pearson avaliou a relação entre o diâmetro dos cardiomiócitos e o Vv da análise por MET. As comparações entre os grupos foram realizadas utilizando o teste exato de Fisher bicaudal e o teste t de Student não pareado. A significância estatística foi definida como p < 0,05.

## Resultados

### Pacientes

O estudo incluiu 18 pacientes com cardiomiopatia *PRKAG2* de cinco famílias não relacionadas ( Tabela 1 ): 10 homens (55,5%), com idade média de 38,5 ± 11,8 anos. A pré-excitação ventricular foi altamente prevalente (15/18; 83,3%), consistentemente associada a uma via fasciculoventricular, e nenhuma via acessória atrioventricular foi identificada. Arritmias atriais ocorreram em 11 pacientes (61,1%), sendo taquicardia atrial em seis (33,3%), flutter atrial em três (16,7%) e fibrilação atrial em dois (11,1%). O grupo controle foi composto por 11 receptores de transplante cardíaco, 8 homens (72,7%), com idade média de 54,9 ± 9,6 anos (idade média dos doadores: 27 ± 8,7 anos, significativamente menor que o grupo *PRKAG2* , p = 0,043). Quatro doadores apresentavam hipertensão sistêmica, estavam em tratamento anti-hipertensivo e demonstraram hipertrofia leve na ecocardiografia. Em conjunto, esses achados destacam a pré-excitação ventricular e as arritmias atriais como características clínicas definidoras da cardiomiopatia *PRKAG2* .

**Tabela 1:** – Características clínicas dos pacientes e perfil histopatológico da biópsia endomiocárdica

Casos	Diagnóstico	Variante	Idade do doador	Idade dos casos	Sexo	Onda delta	Feixe fascículo-ventricular	Taquicardia atrial	Flutter atrial	Fibrilação atrial	Ecocardiograma Hipertrofia do VE	Vacúolos (HE/Manson)	Vacúolos qualitativos	Fibrose intersticial	Desarranjo de miócitos	Inflamação	Diâmetro do miócito (µm^**2**^)	Vv Miofibrilas (%)	Vv Mitocôndrias (%)	Vv Glicogênio (%)
1	PRKAG2	R302Q		47	F	sim/MP	sim*				não	sim	pronunciado	não	não	não	19,20	48,18	27,58	14,85
2	PRKAG2	R302Q		32	F	sim	sim				não	sim	pronunciado	não	não	não	20,16	29,09	33,03	31,82
3	PRKAG2	R302Q		48	F	não	não				não	sim	pronunciado	não	não	não	18,85	x	x	x
4	PRKAG2	R302Q		38	F	sim	sim	paroxístico			não	sim	pronunciado	não	não	não	20,18	39,40	38,18	13,64
5	PRKAG2	R302Q		50	M	sim/MP	sim*			paroxístico	não	sim	pronunciado	não	não	não	17,20	45,10	29,21	16,89
6	PRKAG2	R302Q		39	F	não	não				não	sim	pronunciado	não	não	não	19,32	41,52	27,88	21,21
7	PRKAG2	R302Q		57	M	sim/MP	sim*				sim	sim	pronunciado	não	não	não	25,84	33,64	28,49	31,21
8	PRKAG2	R302Q		55	M	sim/MP	sim*		sim (RF)	persistente	sim	sim	pronunciado	slim leve	não	não	28,38	29,70	25,15	40,31
9	PRKAG2	R302Q		52	M	sim/MP	sim*				sim	sim	pronunciado	não	não	não	17,71	42,42	29,70	24,85
10	PRKAG2	R302Q		32	M	sim	sim				sim	sim	pronunciado	não	não	não	25,08	32,43	30,00	33,34
11	PRKAG2	R302Q		34	M	sim/MP	sim		sim (RF)		sim	sim	pronunciado	não	não	não	26,09	24,85	22,12	38,49
12	PRKAG2	R302Q		36	M	sim/MP	sim*	paroxístico			sim	sim	pronunciado	não	não	não	21,33	39,09	29,40	20,30
13	PRKAG2	R302Q		46	F	sim	sim	paroxístico	sim (RF)		sim	sim	pronunciado	não	não	não	18,24	29,70	24,85	34,85
14	PRKAG2	R302Q		39	M	sim	sim	paroxístico			sim	sim	pronunciado	slim leve	não	não	x	51,22	31,52	10,00
15	PRKAG2	R302Q		30	M	sim/MP	sim			persistente	sim	sim	pronunciado	não	não	não	23,00	32,43	21,52	39,09
16	PRKAG2	H401Q		19	F	sim	sim			paroxístico	sim	sim	pronunciado	não	não	não	21,68	45,76	30,91	15,46
17	PRKAG2	H401Q		18	F	sim	sim				sim	sim	pronunciado	não	não	não	21,35	42,12	36,37	10,31
18	PRKAG2	R302Q		22	M	sim	sim				sim	sim	pronunciado	não	não	não	21,97	34,25	20,61	33,03
19	transplante		24	64	M	não	não				não	não		não	não	slim leve	13,95	54,58	28,05	13,11
20	transplante		31	53	M	não	não				não	sim	leve	não	não	não	16,28	55,46	25,76	9,70
21	transplante		29	55	M	não	não				não	não		slim leve	não	não	13,03	55,28	32,83	2,78
22	transplante		18	52	M	não	não				não	não		não	não	não	16,51	59,88	28,65	5,95
23	transplante		26	61	F	não	não				não	não		não	não	não	16,42	61,79	29,45	4,24
24	transplante		24	56	M	não	não				não	não		não	não	não	17,49	58,79	31,05	3,99
25	transplante		33	33	F	não	não				não	sim	leve	slim leve	não	não	12,77	x	x	x
26	transplante		30	62	M	não	não				sim	não		não	não	não	18,24	x	x	x
27	transplante		15	45	M	não	não				sim	não		slim leve	não	slim leve	21,15	x	x	x
28	transplante		20	68	F	não	não				sim	não		não	não	não	18,18	58,71	30,73	3,97
29	transplante		47	55	M	não	não				sim	não		não	não	não	21,21	49,70	40,30	2,73

*: indica que a pré-excitação desapareceu após o bloqueio atrioventricular; F: feminino; M: masculino; ECG: eletrocardiograma; VE: ventrículo esquerdo; HE: Hematoxilina eosina; MP: marcapasso; RF: ablação por radiofrequência.

### Avaliação da biópsia endomiocárdica

#### Análise histológica

**Figura 1:**
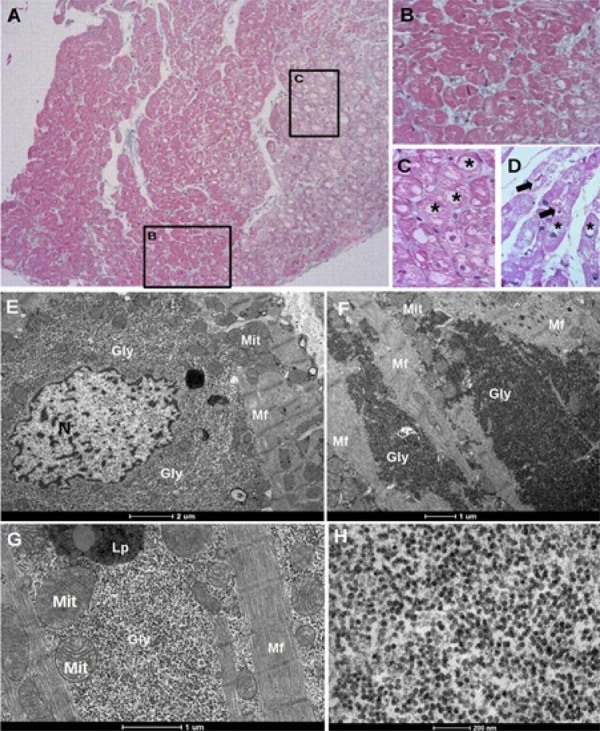
– Achados histopatológicos em um paciente com cardiomiopatia PRKAG2. Cortes histológicos corados com tricrômico de Masson (A, C) revelam arquitetura miocárdica normal, densidade mitocondrial normal, ausência de fibrose ou inflamação e vacuolização pronunciada dos miócitos. Em pontos selecionados, a vacuolização não foi observada (inserção B), em contraste com a maioria das áreas com intensa vacuolização dos cardiomiócitos (inserção C). Micrografias eletrônicas de transmissão (painéis D, G) exibiram grandes quantidades de glicogênio perinuclear (D) e/ou intermiofibrilar (E, F). Os painéis F e G mostram partículas β de glicogênio como pontos pretos com 30 nm; *: vacuolização; Gly: glicogênio; Lp: lipofuscina; Mf: miofibrila; Mit: mitocôndria.

**Figura 2:**
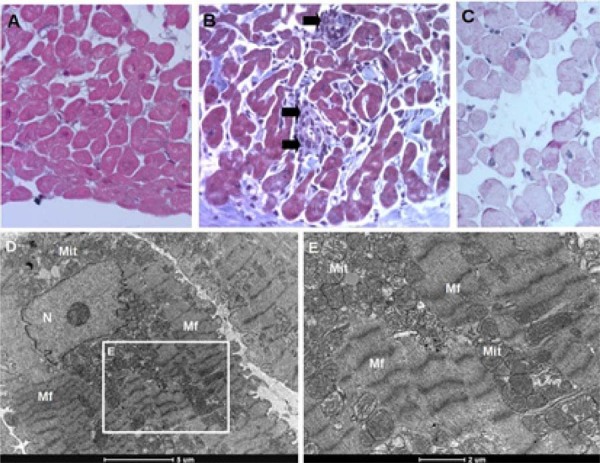
– Achados histopatológicos de biópsia endomiocárdica de um paciente transplantado cardíaco. Cortes corados com A coloração tricrômica de Masson (A, D) revelou arquitetura miocárdica normal, sem fibrose, vacuolização ou inflamação, e densidade mitocondrial normal. Alguns pacientes apresentaram infiltrados inflamatórios (setas) (B) e algumas pequenas áreas com vacuolização (C, D). Apesar da presença desta última, as micrografias eletrônicas de transmissão não revelaram depósitos de glicogênio (E, F). *: vacuolização; Gly: glicogênio; Mf: miofibrila; Mit: mitocôndria.

Pacientes com cardiomiopatia associada ao gene *PRKAG2* apresentaram aumento acentuado dos cardiomiócitos, vacuolização pronunciada e ausência de fibrose intersticial, exceto em dois casos (pacientes 8 e 14) com fibrose leve ( Tabelas 1 e 2 ). Não foi observada infiltração de células inflamatórias ( Figura 1 ). Em contraste, as amostras de controle mostraram infiltrado inflamatório leve em 2 de 11 casos (18,1%), arquitetura normal dos cardiomiócitos, fibrose intersticial leve em 3 casos (27,2%) e vacúolos citosólicos em uma minoria de miócitos em 2 casos (18,1%) ( Figura 2 ). Receptores de transplante cujos doadores apresentavam hipertensão sistêmica exibiram cardiomiócitos maiores do que aqueles cujos doadores eram normotensos (15,2 ± 1,9 µm vs. 19,7 ± 1,7 µm; p = 0,0037).

**Tabela 2:** – Achados histopatológicos do tecido cardíaco

	Transplante de coração (n= 11)	Cardiomiopatia PRKAG2 (n= 17)	Valor p
Fibrose	2/11 (18%)	2/17 (12%)	0,3697^a^
Inflamação	2/11 (18%)	0/17 (0%)	0,1967^a^
Vacuolização	2/11 (18%)	17/17 (100%)	<0,0001^a^
Glicogênio (MET)	0/8 (0%)	17/17 (100%)	<0,0001^a^
Diâmetro dos cardiomiócitos (µm)^#^	16,84 ± 2,86	21,50 ± 3,23	0,0018^b^

^#^Dados apresentados como médias de 12 a 100 cardiomiócitos/paciente; a: Teste exato de Fisher; b: Teste t de Student para amostras independentes; MET: Microscopia eletrônica de transmissão.

#### Microscopia eletrônica de transmissão

Os cardiomiócitos *PRKAG2* apresentaram extenso acúmulo de glicogênio, predominantemente na região perinuclear, com depósitos adicionais nas áreas intermiofibrilares e subsarcolemáticas. Mitocôndrias e sarcômeros pareceram preservados na maioria dos miócitos ( Figura 1D -G). Alguns cardiomiócitos continham partículas de β-glicogênio dispersas entre as mitocôndrias. Grânulos de lipofuscina, correspondentes a resíduos lisossômicos, foram observados tanto nas amostras *PRKAG2* quanto nas amostras controle. Pacientes com a mutação *H401Q* exibiram achados histológicos e ultraestruturais idênticos aos da variante *R302Q* . Notavelmente, nenhuma das amostras controle apresentou acúmulo de glicogênio, mesmo nos casos com leve vacuolização ( Figura 2 ).

**Figura 3:**
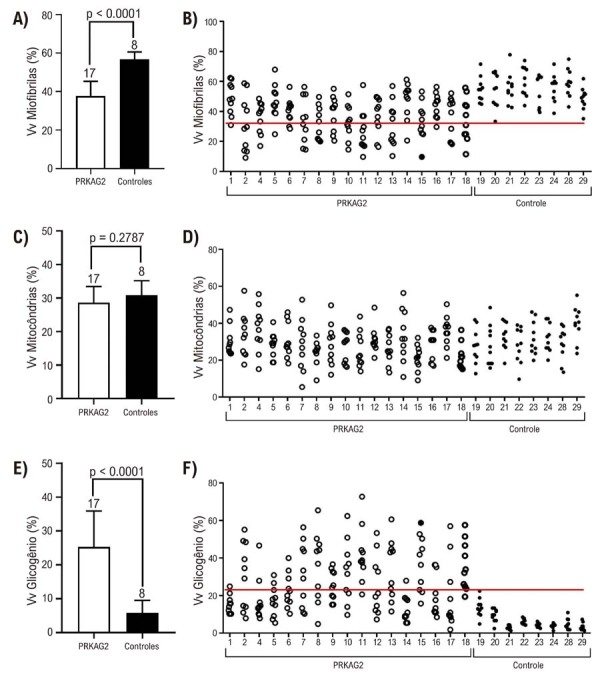
– Densidades volumétricas (Gráfico de densidade óptica (Vv) de miofibrilas (A, B), mitocôndrias (C, D) e glicogênio (E, F) de pacientes com PRKAG2 (barras/círculos brancos) e controles (barras/pontos pretos). Os dados são apresentados como média ± desvio padrão (A, C, E) (número de pacientes descrito acima da barra, teste t de Student não pareado) ou valores individuais de cada paciente (B, D, F) (10 campos por paciente); a linha vermelha indica um limiar nos parâmetros entre os pacientes com PRKAG2 e os controles.

A análise quantitativa por MET revelou um aumento de 6 vezes na densidade de glicogênio (p < 0,0001) em pacientes com *PRKAG2* , uma diminuição de aproximadamente 35% na densidade miofibrilar (p < 0,0001) e nenhuma diferença na densidade mitocondrial entre os grupos ( Figura 3 ). Os valores individuais de densidade volumétrica (Vv) mostraram um limiar claro separando *PRKAG2* dos controles. Os casos de *PRKAG2* apresentaram pelo menos uma imagem de MET com Vv miofibrilas < 30% e Vv glicogênio > 22%. Os casos controle não atingiram esses limiares. Observou-se uma correlação positiva entre o diâmetro do cardiomiócito e o acúmulo de glicogênio, enquanto o conteúdo miofibrilar apresentou uma correlação negativa. A densidade volumétrica mitocondrial não apresentou correlação com o diâmetro do cardiomiócito ( Tabela 3 ).

**Tabela 3:** – Correlação de Pearson entre o diâmetro dos cardiomiócitos e as densidades volumétricas obtidas por MET

	Diâmetro dos cardiomiócitos (µm)		Vv Miofibrilas (%)		Vv Mitocôndrias (%)		Vv Glicogênio (%)	
	Person r	Valor-p	Person r	Valor-p	Person r	Valor-p	Person r	Valor-p
Diâmetro dos cardiomiócitos (µm)			-0,757	< 0,0001	-0,217	0,309	0,706	< 0,001
Vv Miofibrilas (%)					0,338	0,099	-0,924	< 0,0001
Vv Mitocôndrias (%)							-0,616	< 0,001
Vv Glicogênio (%)								

## Dados ausentes

Problemas técnicos durante o processamento por MET impediram uma avaliação confiável em 1 dos 18 pacientes com *PRKAG2* . A análise por microscopia óptica não foi possível para um paciente com *PRKAG2* . Cada paciente teve dois conjuntos de amostras: um para microscopia óptica e outro para MET. No total, 5 dos 58 conjuntos de dados (8,6%) estavam incompletos ( Tabela 1 ).

## Discussão

As características patológicas da cardiomiopatia *PRKAG2* foi descrita em um número limitado de casos, principalmente por meio de BEM, autópsia de vítimas de morte súbita ou exame de corações explantados de receptores de transplante. Esses relatos sugerem que os achados histopatológicos podem variar dependendo do contexto clínico, com alterações mais pronunciadas observadas em corações explantados e em casos de morte súbita.

Dados anatomopatológicos da literatura (Tabela Suplementar 1) indicam que achados morfológicos foram descritos em apenas 22 de 314 pacientes (7%) com cardiomiopatia *PRKAG2* . Ao considerar apenas os casos submetidos a BEM percutânea do VD para fins diagnósticos, esse número é ainda menor, totalizando 14 pacientes (4,5%). O presente estudo é a primeira análise sistemática da BEM do VD em uma grande coorte de pacientes com cardiomiopatia *PRKAG2* , avaliando sua utilidade diagnóstica e a especificidade dos achados em comparação a um grupo controle de receptores de transplante cardíaco cujos doadores não apresentavam a doença.

Os principais resultados do estudo demonstraram que a BEM apresentou alta acurácia diagnóstica em nossa coorte: todos os pacientes com *PRKAG2* positivo (18/18, 100%) exibiram as características morfológicas típicas, incluindo cardiomiócitos aumentados sem desarranjo arquitetônico, vacuolização acentuada e um aumento de 6 vezes na densidade de glicogênio (p < 0,0001). Em contraste, nenhum dos controles apresentou acúmulo de glicogênio e apenas 2/11 exibiram vacuolização focal. Esses resultados indicam que a BEM diferenciou de forma confiável a cardiomiopatia associada ao *PRKAG2* dos controles nesta população estudada.

A vacuolização pronunciada dos cardiomiócitos é uma característica bem documentada da cardiomiopatia *PRKAG2* ,^9 - 14 , 18^ conforme observado em estudos anteriores utilizando coloração H&E ou tricrômico de Masson, tipicamente associada a grânulos PAS-positivos e grandes quantidades de partículas de glicogênio citosólico em MET. Embora a vacuolização também tenha sido observada em dois casos controle, ela foi focal e limitada a uma minoria de cardiomiócitos, provavelmente devido a artefatos de fixação ou processamento ( Figura 2 ). Em contraste, os pacientes com *PRKAG2* exibiram vacuolização generalizada na maioria dos cardiomiócitos.

A explicação predominante para a presença de vacúolos os considera como imagens negativas, um achado indireto resultante da lavagem do glicogênio durante a preparação da lâmina. No entanto, essa suposição não explica completamente a presença de vacúolos em alguns membros do grupo controle. Em um caso controle (caso 20), a MET mostrou claramente a ausência de acúmulo de glicogênio no citosol ( Tabela 1 ). Vale ressaltar que alguns indivíduos normais podem apresentar maior densidade volumétrica de glicogênio ( Figura 3F ), porém, não demonstram o acúmulo de glicogênio observado em pacientes com *PRKAG2* ( Figura 1D -G vs. Figura 2E F).

A hipertrofia cardíaca é uma característica marcante da cardiomiopatia associada ao gene *PRKAG2* . Recentemente, relatamos a utilidade diagnóstica da ecocardiografia transtorácica, que identificou hipertrofia ventricular esquerda em 25/30 (86%) e hipertrofia ventricular direita em 26/30 (90%) portadores de variantes patogênicas do *PRKAG2* .^27^ No entanto, em nível celular, a hipertrofia é tipicamente caracterizada por um aumento no número de unidades sarcoméricas dentro dos cardiomiócitos. O aumento do diâmetro dos cardiomiócitos, por si só, não é diagnóstico de hipertrofia, visto que a expansão celular pode resultar de diversos fatores, incluindo o armazenamento excessivo de substâncias intracelulares como glicogênio, lipídios e água, que podem se manifestar como hipertrofia ventricular em exames de imagem cardíaca.

Estudos recentes utilizando cardiomiócitos derivados de células-tronco pluripotentes induzidas humanas (hiPSC-CMs)^28 , 29^ mostraram que mutações no gene *PRKAG2* ( *R302Q* e *N488I* ) induzem o aumento do tamanho celular devido ao acúmulo de glicogênio, impulsionado por um aumento persistente e inadequado na atividade da AMPK. Notavelmente, a inibição da atividade da AMPK com o composto C reduziu o armazenamento de glicogênio e reverteu a hipertrofia detectada por imagem.^29^ A subunidade β da AMPK contém um domínio regulatório de ligação ao glicogênio, permitindo que a AMPK do tipo selvagem funcione como um sensor de glicogênio,^30^ enquanto a microscopia de imunogold mostrou que as subunidades da AMPK se co-localizam com partículas de glicogênio, com a subunidade β1 posicionada na periferia das rosetas de glicogênio.^31^

Em nosso estudo, observamos uma correlação positiva entre o aumento do tamanho dos cardiomiócitos e o acúmulo intracelular de glicogênio, juntamente com uma correlação inversa entre a densidade volumétrica das miofibrilas e o diâmetro dos cardiomiócitos. Como a densidade volumétrica é uma medida relativa, não podemos descartar um possível aumento no conteúdo de miofibrilas no contexto de cardiomiócitos maiores. Curiosamente, um estudo utilizando um modelo de camundongo transgênico com superexpressão cardíaca do mutante *N488I PRKAG2* , combinado com uma mutação de inserção no gene do glicogênio sintase muscular (GYS1) – que inibe a atividade do glicogênio sintase estimulada pela glicose-6-fosfatase – conseguiu resgatar o fenótipo de armazenamento de glicogênio e a pré-excitação ventricular. No entanto, isso não afetou a hipertrofia cardíaca ou o aumento do tamanho dos cardiomiócitos, sugerindo que mecanismos além do acúmulo de glicogênio contribuem para a hipertrofia na cardiomiopatia *PRKAG2* .^32^

Uma descoberta importante do nosso estudo foi que mesmo pacientes com *PRKAG2* sem hipertrofia cardíaca aparente apresentaram alterações morfológicas, incluindo aumento do conteúdo de glicogênio, ausência de inflamação e fibrose mínima ou ausente ( Tabela 1 ). Embora a vacuolização dos cardiomiócitos ocorra em corações normais e em outras doenças, ela é muito mais pronunciada e disseminada na cardiomiopatia associada à *PRKAG2* ( Figura 1 ). Além disso, os grandes depósitos de glicogênio identificados na MET foram altamente específicos para *PRKAG2* e ausentes nos controles. Exceções incluem casos com fibrose intersticial e desarranjo arquitetônico, relatados em variantes raras do *PRKAG2* , como *K485E* e *E506K* .^3 , 6^ Os achados em corações explantados ou autopsiados em doença avançada também diferem, mostrando mais fibrose,^7 , 8 , 14 , 16 - 19^desarranjo arquitetônico^16 , 17^e degeneração ocasional de miócitos.^14 , 17^ Essas características de “estágio final” também foram relatadas em variantes comuns do *PRKAG2* , como *R302Q* e *N488I* .^16 , 17^

Neste estudo, a correlação genótipo-fenótipo foi consistente em nossa coorte. Todos os membros da família de um probando com uma variante no gene *PRKAG2* e características clínicas típicas também eram portadores da mesma variante, em consonância com relatos anteriores que descrevem a forte associação entre mutações no *PRKAG2* e esse fenótipo. Essa concordância completa em nossa série reforça a robustez da associação genético-clínica, evitando generalizações excessivas para além da população estudada.

Outras condições precisam ser diferenciadas da cardiomiopatia *PRKAG2* . A cardiotoxicidade induzida pela hidroxicloroquina, tipicamente resultante do uso prolongado para doenças do tecido conjuntivo, também se apresenta com vacuolização pronunciada dos miócitos, que se estende ao músculo esquelético. A BEM nesse contexto mostra fibrose intersticial focal sem inflamação. A microscopia eletrônica revela extensas inclusões lisossômicas, corpos mieloides e inclusões curvilíneas, mas sem acúmulo de glicogênio.^33^ A doença de Danon (mutação *LAMP2* ), que exibe um fenótipo cardíaco grave predominante em homens jovens (geralmente abaixo de 20 anos),^34^ met um perfil microscópico distinto. O exame post-mortem de dois corações mostrou hipertrofia maciça, desarranjo substancial dos miócitos e fibrose, com aglomerados proeminentes de miócitos vacuolizados e inclusões de material granular amorfo em algumas células dentro das regiões cicatriciais. A doença de Anderson-Fabry, que pode apresentar um fenótipo cardíaco dominante, está associada ao envolvimento multissistêmico.^35^ Histologicamente, apresenta extensa vacuolização dos cardiomiócitos, fibrose e desarranjo dos miócitos, particularmente no VD, com necrose ocasional de miócitos e substituição por macrófagos espumosos. No entanto, não se observa acúmulo de glicogênio. Além disso, as cardiomiopatias mitocondriais, como as associadas à mutação *3243A>G* , podem apresentar um fenótipo semelhante ao da cardiomiopatia hipertrófica (CMH), frequentemente diagnosticadas erroneamente como cardiomiopatia isolada. Esses pacientes podem não apresentar episódios semelhantes a acidente vascular cerebral (síndrome MELAS), e a MET tipicamente revela proliferação mitocondrial.^36^

### Limitações

O grupo de controle consistiu em pacientes transplantados cardíacos cujos corações doadores eram significativamente mais jovens do que aqueles com cardiomiopatia *PRKAG2* . No entanto, não se espera que as alterações patológicas características da cardiomiopatia *PRKAG2* surjam do envelhecimento normal. Alguns dados foram perdidos devido a problemas durante a fixação ou o processamento das amostras (para MET: três casos no grupo controle e um no grupo com cardiomiopatia *PRKAG2* ; para microscopia óptica: um caso no grupo com cardiomiopatia *PRKAG2* ). Considerando que havia apenas 2/36 conjuntos de dados faltantes (5,5%) no grupo com cardiomiopatia *PRKAG2* , é improvável que isso tenha tido um impacto significativo em nossos resultados.

## Conclusão

Os achados da BEM percutânea do VD revelam alterações morfológicas características, diagnósticas da cardiomiopatia associada ao gene *PRKAG2* , em todos os genótipos dos pacientes positivos. A MET desempenha um papel importante na detecção do acúmulo substancial de glicogênio nos cardiomiócitos. As anormalidades histopatológicas e ultraestruturais descritas neste estudo não foram observadas nas amostras de controle. Nossos achados sugerem que a BEM, associada à microscopia óptica e eletrônica, pode auxiliar no diagnóstico da cardiomiopatia associada ao gene *PRKAG2* . Estudos adicionais são necessários para validar a microscopia como ferramenta diagnóstica para essa condição.

## Material suplementar

Supplementary Table 1

## References

[1] Yavari A, Sarma D, Sternick EB (2018). Methods Mol Biol.

[2] Arad M, Benson DW, Perez-Atayde AR, McKenna WJ, Sparks EA, Kanter RJ (2002). Constitutively Active AMP Kinase Mutations Cause Glycogen Storage Disease Mimicking Hypertrophic Cardiomyopathy. J Clin Invest.

[3] Bayrak F, Komurcu-Bayrak E, Mutlu B, Kahveci G, Basaran Y, Erginel-Unaltuna N (2006). Ventricular Pre-Excitation and Cardiac Hypertrophy Mimicking Hypertrophic Cardiomyopathy in a Turkish Family with a Novel PRKAG2 Mutation. Eur J Heart Fail.

[4] Kelly BP, Russell MW, Hennessy JR, Ensing GJ (2009). Severe Hypertrophic Cardiomyopathy in an Infant with a Novel PRKAG2 Gene Mutation: Potential Differences between Infantile and Adult Onset Presentation. Pediatr Cardiol.

[5] Sternick EB, Oliva A, Gerken LM, Magalhães L, Scarpelli R, Correia FS (2011). Clinical, Electrocardiographic, and Electrophysiologic Characteristics of Patients with a Fasciculoventricular Pathway: The Role of PRKAG2 Mutation. Heart Rhythm.

[6] Liu Y, Bai R, Wang L, Zhang C, Zhao R, Wan D (2013). Identification of a Novel de Novo Mutation Associated with PRKAG2 Cardiac Syndrome and Early Onset of Heart Failure. PLoS One.

[7] Thevenon J, Laurent G, Ader F, Laforêt P, Klug D, Pentiah AD (2017). High Prevalence of Arrhythmic and Myocardial Complications in Patients with Cardiac Glycogenosis due to PRKAG2 Mutations. Europace.

[8] Yogasundaram H, Paterson ID, Graham M, Sergi C, Oudit GY (2016). Glycogen Storage Disease because of a PRKAG2 Mutation Causing Severe Biventricular Hypertrophy and High-Grade Atrio-Ventricular Block. Circ Heart Fail.

[9] Sternick EB, Araújo SA, Camargos ERS, Brasileiro G (2016). Atrial Pathology Findings in a Patient with PRKAG2 Cardiomyopathy and Persistent Atrial Fibrillation. Circ Arrhythm Electrophysiol.

[10] Banankhah P, Fishbein GA, Dota A, Ardehali R (2018). Cardiac Manifestations of PRKAG2 Mutation. BMC Med Genet.

[11] Sri A, Daubeney P, Prasad S, Baksi J, Kinali M, Voges I (2019). A Case Series on Cardiac and Skeletal Involvement in Two Families with PRKAG2 Mutations. Case Rep Pediatr.

[12] Siqueira MHS, Honorato-Sampaio K, Dias GM, Wilson JR, Yavari A, Brasileiro G (2020). Sudden Death Associated with a Novel H401Q PRKAG2 Mutation. Europace.

[13] Lopez-Sainz A, Dominguez F, Lopes LR, Ochoa JP, Barriales-Villa R, Climent V (2020). Clinical Features and Natural History of PRKAG2 Variant Cardiac Glycogenosis. J Am Coll Cardiol.

[14] Hu D, Hu D, Liu L, Barr D, Liu Y, Balderrabano-Saucedo N (2020). Identification, Clinical Manifestation and Structural Mechanisms of Mutations in AMPK Associated Cardiac Glycogen Storage Disease. EBioMedicine.

[15] Frustaci A, Russo MA, Chimenti C (2013). Diagnostic Contribution of Left Ventricular Endomyocardial Biopsy in Patients with Clinical Phenotype of Hypertrophic Cardiomyopathy. Hum Pathol.

[16] Murphy RT, Mogensen J, McGarry K, Bahl A, Evans A, Osman E (2005). Adenosine Monophosphate-Activated Protein Kinase Disease Mimicks Hypertrophic Cardiomyopathy and Wolff-Parkinson-White Syndrome: Natural History. J Am Coll Cardiol.

[17] Tan HL, van der Wal AC, Campian ME, Kruyswijk HH, ten Hove Jansen B, van Doorn DJ (2008). Nodoventricular Accessory Pathways in PRKAG2-Dependent Familial Preexcitation Syndrome Reveal a Disorder in Cardiac Development. Circ Arrhythm Electrophysiol.

[18] van der Steld LP, Campuzano O, Pérez-Serra A, Zamorano MMB, Matos SS, Brugada R (2017). Wolff-Parkinson-White Syndrome with Ventricular Hypertrophy in a Brazilian Family. Am J Case Rep.

[19] Pöyhönen P, Hiippala A, Ollila L, Kaasalainen T, Hänninen H, Heliö T (2015). Cardiovascular Magnetic Resonance Findings in Patients with PRKAG2 Gene Mutations. J Cardiovasc Magn Reson.

[20] Gollob MH, Green MS, Tang AS, Gollob T, Karibe A, Ali Hassan AS (2001). Identification of a Gene Responsible for Familial Wolff-Parkinson-White Syndrome. N Engl J Med.

[21] Blair E, Redwood C, Ashrafian H, Oliveira M, Broxholme J, Kerr B (2001). Mutations in the Gamma(2) Subunit of AMP-Activated Protein Kinase Cause Familial Hypertrophic Cardiomyopathy: Evidence for the Central Role of Energy Compromise in Disease Pathogenesis. Hum Mol Genet.

[22] Cooper LT, Baughman KL, Feldman AM, Frustaci A, Jessup M, Kuhl U (2007). The Role of Endomyocardial Biopsy in the Management of Cardiovascular Disease: A Scientific Statement from the American Heart Association, the American College of Cardiology, and the European Society of Cardiology. Circulation.

[23] From AM, Maleszewski JJ, Rihal CS (2011). Current Status of Endomyocardial Biopsy. Mayo Clin Proc.

[24] Richards S, Aziz N, Bale S, Bick D, Das S, Gastier-Foster J (2015). Standards and Guidelines for the Interpretation of Sequence Variants: A Joint Consensus Recommendation of the American College of Medical Genetics and Genomics and the Association for Molecular Pathology. Genet Med.

[25] Walsh R, Thomson KL, Ware JS, Funke BH, Woodley J, McGuire KJ (2017). Reassessment of Mendelian Gene Pathogenicity using 7,855 Cardiomyopathy Cases and 60,706 Reference Samples. Genet Med.

[26] Cantuária VL, Rodrigues CM, Dias IR, Ottone VO, Costa BO, Godinho LF (2025). Intense Caloric Restriction from Birth Protects the Heart Against Ischemia/Reperfusion Injury and Reduces Reactive Oxygen Species in Ovariectomized Rats. Antioxidants.

[27] Pena JLB, Santos WC, Siqueira MHA, Sampaio IH, Moura ICG, Sternick EB (2021). Glycogen Storage Cardiomyopathy (PRKAG2): Diagnostic Findings of Standard and Advanced Echocardiography Techniques. Eur Heart J Cardiovasc Imaging.

[28] Hinson JT, Chopra A, Lowe A, Sheng CC, Gupta RM, Kuppusamy R (2016). Integrative Analysis of PRKAG2 Cardiomyopathy iPS and Microtissue Models Identifies AMPK as a Regulator of Metabolism, Survival, and Fibrosis. Cell Rep.

[29] Zhan Y, Sun X, Li B, Cai H, Xu C, Liang Q (2018). Establishment of a PRKAG2 Cardiac Syndrome Disease Model and Mechanism Study using Human Induced Pluripotent Stem Cells. J Mol Cell Cardiol.

[30] McBride A, Ghilagaber S, Nikolaev A, Hardie DG (2009). The Glycogen-Binding Domain on the AMPK Beta Subunit Allows the Kinase to Act as a Glycogen Sensor. Cell Metab.

[31] Bendayan M, Londono I, Kemp BE, Hardie GD, Ruderman N, Prentki M (2009). Association of AMP-Activated Protein Kinase Subunits with Glycogen Particles as Revealed in Situ by Immunoelectron Microscopy. J Histochem Cytochem.

[32] Kim M, Hunter RW, Garcia-Menendez L, Gong G, Yang YY, Kolwicz SC (2014). Mutation in the ?2-Subunit of AMP-Activated Protein Kinase Stimulates Cardiomyocyte Proliferation and Hypertrophy Independent of Glycogen Storage. Circ Res.

[33] Nadeem U, Raafey M, Kim G, Treger J, Pytel P, Husain AN (2021). Chloroquine- and Hydroxychloroquine-Induced Cardiomyopathy: A Case Report and Brief Literature Review. Am J Clin Pathol.

[34] Maron BJ, Roberts WC, Arad M, Haas TS, Spirito P, Wright GB (2009). Clinical Outcome and Phenotypic Expression in LAMP2 Cardiomyopathy. JAMA.

[35] Sheppard MN, Cane P, Florio R, Kavantzas N, Close L, Shah J (2010). A Detailed Pathologic Examination of Heart Tissue from three Older Patients with Anderson-Fabry Disease on Enzyme Replacement Therapy. Cardiovasc Pathol.

[36] Hiraki N, Tanaka TD, Yoshimura M (2022). A Man with Left Ventricular Hypertrophy. JAMA Cardiol.

