# Supplementary material for: Cardiomiopatia PRKAG2: Um Estudo Caso-Controle sobre o Rendimento Diagnóstico da Histopatologia e da Análise Ultraestrutural da Biópsia Endomiocárdica
Source: Arq Bras Cardiol. 2026 Feb 27;123(2):e20240616. [Article in Portuguese] doi: 10.36660/abc.20240616 (PMC13128181; doi:10.36660/abc.20240616)
Supplement: Supplementary Table 1 [file 0066-782x-abc-123-2-e20240616-suppl01.pdf]

Supplementary Table 1 – Anatomopathological data from the literature

	Manuscript	total number of patients	Source	Cases with biopsy or autopsy	Patient identification	Age (years)	Sex	Variant	Heart weight in autopsy (g)	Vacuolization (H&E)	Glycogen (PAS+)	hypertrophy	Fibrosis (Masson's trychrome)	Inflammation	Myocite disarray or degeneration	Ultrastructural examination of heart
1	Arad M, et al., J Clin Invest 2002;109:357–362	70	Autopsy (sudden death)	1	-	26	-	N488I	-	pronounced	yes	yes	minimal interstitial fibrosis	no	no	-
			RV EMB	2	II:2	39	-	T400N	-	profound	yes	yes	no	no	no	increased glycogen
2	Murphy RT, et al, JACC 2005;45:922-30	45	Autopsy (sudden death)	1	?	27	-	N488I	-	extensive	yes	yes	coarse interstitial fibrosis	no	minimal disarray	non specific degenerative changes in mithocondria. Increased glycogen
3	Bayrak F, et al., Eur J Heart Fail 2006;8:712-5	8	RV EMB	1	IV.1	19	F	E506K	-	pronounced	-	yes	minimal interstitial fibrosis	no	no	-
4	Tan HL, et al., Circ Arrhythm Electrophysiol 2008;1:276-81	10	Autopsy (sudden death)	1	III:8	42	F	R302Q	540	slight	yes	yes	slight interstitial fibrosis	no	extensive myocardial disarray (20% LV mass)	-
5	Kelly BP, et al., Pediatr Cardiol 2009;30:1176-9	1	RV EMB	1	-	1.5	M	E506Q	-	no	no	no	no	no	no	normal amount of glycogen
6	Sternick EB, et al., Heart Rhythm 2011;8:58-64	10	RV EMB	1	I F-3	20	M	R302Q	-	profound	yes	yes	no	no	no	-
7	Liu Y, et al., PLoS ONE 2013;8(5):e64603	1	RV EMB	1	II:2	19	M	K485E	-	profound	yes	yes	intersticial fibrosis	no	prominent focal myocite disarray	increased glycogen
8	Poyhonen P, et al., J Cardiovasc Mag Res 2015;17:89	6	Heart explant - heart transplant	1	I:1	24	M	H344P	-	pronounced	yes	yes	focal	no	no	increased glycogen
9	Thevenon J, et al., Europace 2016;10.1093/europace/euw067	34	RV EMB	1	II:1 F-1	-	F	R302Q	-	pronounced	yes	yes	no	no	no	-

			RV EMB	2	II:1 F-4	-	F	R302Q	-	no	no	yes	no	no	no	-
			Heart explant - heart transplant	3	III:1 F-9	-	F	H530R	373	pronounced	-	yes	interstitial fibrosis	no	myofibrillar disorganization	-
10	Yogasundaran H, et al., Circ Heart Fail 2016;9:e003367	1	RV EMB	1	-	31	M	R302Q		pronounced	yes	yes	interstitial fibrosis	no	no	increased glycogen and mitochondrial changes
11	Back Sternick E, et al., Circ Arrhythm Electrophysiol 2016;9:e004455	1	RV EMB	1	-	46	M	R302Q	-	pronounced	yes	yes	no	no	no	large cytosolic pools of glycogen
			R Atrial EMB	1	-	-	-	-	-	pronounced	yes	yes	no	no	no	large cytosolic pools of glycogen
12	Van der Steld LP, et al., Am J Case Rep 2017;18:766-77	19	Autopsy (sudden death)	1	-	22	M	K290I	786	severe	yes	biventricular	interstitial fibrosis	no	no	increased glycogen
13	Bhanankha P, et al., BMC Med Genet 2018;19:1-4	1	RV EMB	1	-	23	F	R302Q	-	pronounced	yes	yes	no	no	no	marked cytoplasmic glycogen accumulation
14	Albernaz Siqueira MH et al., Europace 2020. doi:10.1093/europace/euaa/014/58 111872	5	RV EMB	1	III.1	20	F	H401Q	-	pronounced	yes	yes	no	no	no	large cytosolic pools of glycogen
			RV EMB	2	III.3	18	F	H401Q		pronounced	yes	yes	no	no	no	large cytosolic pools of glycogen
15	Lopes-Sainz A et al., JACC 2020;76(2):186-97	90	RV EMB	1	-	-	-	R302Q		pronounced	yes	yes	no	no	no	-
16	Hu D, et al., EBioMedicine 2020;54:102723	12	Autopsy	1	proband12	22	M	K485E	1043	pronounced	-	marked	intersticial fibrosis	no	myocite disarray	large amounts of glycogen
			RV EMB	2	proband8	24	M	R302Q		pronounced	-	yes	no	no	partial degeneration	-
