# Supplementary material for: Cardiomiopatia PRKAG2: Um Estudo Caso-Controle sobre o Rendimento Diagnóstico da Histopatologia e da Análise Ultraestrutural da Biópsia Endomiocárdica
Source: Arq Bras Cardiol. 2026 Feb 27;123(2):e20240616. [Article in Portuguese] doi: 10.36660/abc.20240616 (PMC13128181; doi:10.36660/abc.20240616)
Supplement: Translation

# Translation: PRKAG2 Cardiomyopathy: A Case-Control Study on the Diagnostic Yield Of Histopathology and Ultrastructural Analysis from Endomyocardial Biopsy

**DOI:** 10.36660/abc.20240616i

**Authors:** Kinulpe Honorato-Sampaio, Carla de Oliveira, Stanley de Almeida Araújo, Frederico Soares Correa, Glauber Monteiro Dias, Antônio Fernandino de Castro Bahia, Vinícius Lopes Cantuária, Arash Yavari, Maria da Consolação Vieira Moreira, José Luiz Barros Pena, Geraldo Brasileiro, Eduardo Back Sternick

**Affiliations:** 1 Brazil Faculdade de Medicina, Universidade Federal do Vale do Jequitinhonha e Mucuri, Diamantina, MG – Brazil; 2 Brazil Faculdade de Ciências Médicas, Belo Horizonte, MG – Brazil; 3 Brazil Hospital das Clínicas - Universidade Federal de Minas Gerais, Belo Horizonte, MG – Brazil; 4 Brazil Hospital Biocor, Rede D’Or São Luiz, Nova Lima, MG – Brazil; 5 Brazil Laboratório de Biologia Celular e Tecidual, Centro de Biociências e Biotecnologia - Universidade Estadual do Norte Fluminense Darcy Ribeiro, Campos dos Goytacazes, RJ – Brazil; 6 Brazil Departamento de Cardiologia - Hospital Nossa Senhora das Graças, Sete Lagoas, MG – Brazil; 7 United Kingdom Experimental Therapeutics and Division of Cardiovascular Medicine, Radcliffe Department of Medicine, University of Oxford, Oxford – United Kingdom; 8 Brazil Departamento de Clínica Médica - Faculdade de Medicina, Universidade Federal de Minas Gerais, Belo Horizonte, MG – Brazil; 9 Brazil Departamento de Anatomia Patológica, Faculdade de Medicina - Universidade Federal de Minas Gerais, Belo Horizonte, MG – Brazil; 10 Brazil Hospital Felício Rocho, Belo Horizonte, MG – Brazil

**Keywords:** AMP-Activated Protein Kinases, Cardiomegaly, Microscopy, Electron, Transmission

## Abstract

**Background:**

The histopathological features of PRKAG2 cardiomyopathy have been reported in a fragmentary manner.

**Objective:**

We aimed to systematically evaluate the cardiac pathological features of PRKAG2 cardiomyopathy in a large patient cohort and assess their diagnostic potential compared to genetic sequencing.

**Methods:**

We conducted an observational, cross-sectional, case-control study including 18 patients with PRKAG2 cardiomyopathy and 11 heart transplant recipients as controls. All patients underwent percutaneous right ventricular endomyocardial biopsy. Tissue samples were analyzed using H&E staining, Periodic Acid-Schiff staining for glycogen, Masson’s trichrome for fibrosis, and ultrastructural assessment by transmission electron microscopy. Statistical significance was set at p < 0.05 for all analyses.

**Results:**

PRKAG2 cardiomyopathy hearts exhibited significant cardiomyocyte enlargement, normal-appearing mitochondria, extensive vacuolization of most myofibers, minimal interstitial fibrosis (only two patients had mild fibrosis), and no inflammatory cell infiltration. Transmission electron microscopy revealed abundant cytosolic glycogen, primarily in the perinuclear region, with additional deposits in intermyofibrillar and subsarcolemmal areas. This pronounced glycogen accumulation, consistently observed in all PRKAG2 patients, was absent in controls.

**Conclusion:**

Histological and ultrastructural examination of right ventricular endomyocardial biopsy samples reveals a distinct set of features that strongly suggest PRKAG2 cardiomyopathy.

## Introduction

Mutations in the *PRKAG2* gene, which encodes the regulatory γ2 subunit of AMP-activated protein kinase, lead to a highly penetrant phenotype of ventricular hypertrophy, pre-excitation, atrial tachyarrhythmias, progressive sinus bradycardia, and cardiac conduction abnormalities.^1^The hallmark pathological feature – abnormal cardiomyocyte glycogen accumulation – initially led to its classification as a glycogen storage cardiomyopathy and a phenocopy of sarcomeric hypertrophic cardiomyopathy.^2^

Since its first descriptions, the morphological characteristics of *PRKAG2* cardiomyopathy have been primarily reported using right ventricular septal endomyocardial biopsy (EMB) samples,^2 - 15^a limited number of autopsies from patients who died suddenly,^2 , 14 , 16 , 17 , 18^and explanted hearts from transplant recipients.^7 , 19^Pathological findings appear to depend on the timing of the biopsy within the disease’s natural history, with more pronounced abnormalities observed in patients with severe clinical manifestations.^1 - 19^For instance, explanted hearts from transplant recipients with end-stage heart failure often exhibit distinct features compared to those of young patients who succumbed to sudden cardiac death.

Since the identification of the *PRKAG2* gene,^20 , 21^genetic sequencing has become the gold standard for diagnosis. However, the potential role of cardiac pathology assessment has never been systematically evaluated. Right ventricular (RV) EMB is a safe diagnostic tool that was widely used in the evaluation of suspected glycogen storage cardiomyopathies before genetic testing became widely available.^22 , 23^In Brazil and other lower-income countries, access to genetic testing remains limited, whereas EMB is covered by the public healthcare system. It is unclear, however, whether EMB provides diagnostic accuracy comparable to genetic sequencing, which has a reported yield of 100%.

Accordingly, we conducted a cross-sectional case-control study to systematically assess the histological and ultrastructural features of *PRKAG2* cardiomyopathy using: (1) a cohort of 18 patients from five families carrying the *R302Q* (the most frequently reported pathogenic variant) and *H401Q* variants of the *PRKAG2* gene; (2) a control group of 11 structurally normal transplanted hearts. Histological and ultrastructural analysis of RV-EMB revealed a set of features suggestive of *PRKAG2* cardiomyopathy, including cardiomyocyte enlargement, pronounced vacuolization, absence of interstitial fibrosis, substantial glycogen accumulation, and preserved mitochondrial volumetric density (Central Illustration).

## Methods

### Study design

This prospective observational case-control study was approved by the Scientific Ethics Committee of Universidade Federal de Minas Gerais (SISNEP CAAE: 0511.0.203.418-11). Written informed consent for EMB was obtained from all participants. The study was designed to assess the diagnostic performance of EMB in patients with genetically confirmed *PRKAG2* cardiomyopathy, using transplant recipients as controls.

### Study population

The case group comprised 18 patients with pathogenic *PRKAG2* variants (16 with the *R302Q* mutation and two with the *H401Q* mutation, recently reported by our group^12^). The control group consisted of 11 heart transplant recipients with no clinical evidence of graft rejection who underwent routine EMB within 10 days post-transplant for rejection surveillance. Transplanted hearts were structurally and electrocardiographically normal based on echocardiography and electrocardiography.

### Genetic analysis

Genomic DNA from *PRKAG2* cardiomyopathy patients, isolated from whole blood leukocytes, underwent sequencing using a 52-gene cardiomyopathy panel. A genomic library was prepared with an AmpliSeq custom panel and sequenced using a PGM Ion Torrent system. Sequences were aligned to the hg19 reference genome, and nucleotide variants were filtered following the American College of Medical Genetics and Genomics and Association for Molecular Pathology guidelines.^24 , 25^Family members underwent mutation-specific screening by Sanger sequencing using an ABI 3500XL genetic analyzer (Thermo Fisher Scientific, Waltham, MA, USA).

### Endomyocardial biopsy

Under local anesthesia, an experienced operator performed EMB using a Cook^®^myocardial biopsy forceps (Bloomington, IN, USA), targeting the right interventricular septum to obtain five tissue samples. Three fragments were fixed in 10% formalin for 12 hours at room temperature for light microscopy. The remaining two were fixed in Karnovsky’s solution (2.5% glutaraldehyde and 2% paraformaldehyde in 0.1M cacodylate buffer, pH 7.4) overnight at 4°C for electron microscopy. No patient experienced complications.

### Light microscopy

Formalin-fixed EMB fragments were embedded in paraffin, sectioned (2 µm), and stained with hematoxylin-eosin (H&E), periodic acid-Schiff (PAS), and Masson’s trichrome. Cardiomyocyte diameter (≥12 myocytes per patient) was measured using ImageJ software v1.49 (National Institutes of Health, USA). Photomicrographs were taken at ×400 magnification using an Axio Imager Z2 - Apotome 2 microscope (Zeiss, USA).

### Transmission electron microscopy

Samples were post-fixed in 1% OsO_4_, stained overnight with 2% uranyl acetate, dehydrated in ethanol, and embedded in Epon 812 (EMS, USA). Ultrathin sections (50 nm) were cut using an ultramicrotome with a diamond knife, mounted on copper grids, and stained with Reynolds lead citrate. Transmission electron microscopy (TEM) was performed at 80 kV using a FEI Tecnai G2-12 Spirit, equipped with a SIS-MegaView 3 CCD camera. Micrographs were taken at ×9,900 magnification. Images were randomly selected from central cardiomyocyte regions and analyzed using ImageJ software. Volume densities (Vv) of glycogen, mitochondria, and myofibrils were determined using the classic point counting method with a 165-point grid (500 × 500 nm) projected onto each image, following Lopes Cantuária et al.^26^At least 10 cardiomyocytes per patient underwent morphometric analysis.

### Statistical analysis

As *PRKAG2* cardiomyopathy is an extremely rare disease, the study sample was defined by convenience, including all eligible patients available during the study period. Microscopy images were analyzed in a blinded manner by two investigators. Any discrepancies were resolved by consensus with a third investigator. Data were analyzed using GraphPad Prism v6.1 (GraphPad Software, USA) and presented as mean ± standard deviation (SD). Categorical variables were presented as absolute and relative frequencies. Morphometric data were normally distributed (Shapiro-Wilk test). Pearson’s correlation assessed the relationship between cardiomyocyte diameter and Vv from TEM analysis. Group comparisons were performed using two-tailed Fisher’s exact test and Student’s unpaired t-test. Statistical significance was set at p < 0.05.

## Results

### Patients

The study included 18 patients with *PRKAG2* cardiomyopathy from five unrelated families ( Table 1 ): 10 males (55.5%), mean age 38.5 ± 11.8 years. Ventricular pre-excitation was highly prevalent (15/18; 83.3%), consistently associated with a fasciculo-ventricular pathway, and no atrioventricular accessory pathway was identified. Atrial arrhythmias occurred in 11 patients (61.1%), comprising atrial tachycardia in six (33.3%), atrial flutter in three (16.7%), and atrial fibrillation in two (11.1%). The control group comprised 11 heart transplant recipients, 8 males (72.7%), with a mean age of 54.9 ± 9.6 years (donor mean age: 27 ± 8.7 years, significantly younger than the *PRKAG2* group, p = 0.043). Four donors had systemic hypertension, were on antihypertensive therapy, and showed mild hypertrophy on echocardiography. Together, these findings highlight ventricular pre-excitation and atrial arrhythmias as defining clinical features of *PRKAG2* cardiomyopathy.

**Table 1:** – Clinical characteristics of patients and histopathological profile of endomyocardial biopsy

Cases	Diagnosis	Variant	Age - donor	Age - cases	Gender	ECG Delta wave	Fasciculo ventricular pathway	Atrial tachycardia	Atrial flutter	Atrial fibrillation	Echocardiogram LV Hypertrophy	vacuoles (HE/Masson)	vacuoles qualitative	Interstitial fibrosis	Myocyte disarray	Inflammation	Myocyte diameter (um^**2**^)	Vv Myofibrils (%)	Vv Mitochondria (%)	Vv Glycogen (%)
1	PRKAG2	R302Q		47	F	yes/ PM	yes*				no	yes	pronounced	no	no	no	19.20	48.18	27.58	14.85
2	PRKAG2	R302Q		32	F	yes	yes				no	yes	pronounced	no	no	no	20.16	29.09	33.03	31.82
3	PRKAG2	R302Q		48	F	no	no				no	yes	pronounced	no	no	no	18.85	x	x	x
4	PRKAG2	R302Q		38	F	yes	yes	paroxysmal			no	yes	pronounced	no	no	no	20.18	39.40	38.18	13.64
5	PRKAG2	R302Q		50	M	yes/ PM	yes*			paroxysmal	no	yes	pronounced	no	no	no	17.20	45.10	29.21	16.89
6	PRKAG2	R302Q		39	F	no	no				no	yes	pronounced	no	no	no	19.32	41.52	27.88	21.21
7	PRKAG2	R302Q		57	M	yes/ PM	yes*				yes	yes	pronounced	no	no	no	25.84	33.64	28.49	31.21
8	PRKAG2	R302Q		55	M	yes/ PM	yes*		yes (RF)	persistent	yes	yes	pronounced	yes mild	no	no	28.38	29.70	25.15	40.31
9	PRKAG2	R302Q		52	M	yes/ PM	yes*				yes	yes	pronounced	no	no	no	17.71	42.42	29.70	24.85
10	PRKAG2	R302Q		32	M	yes	yes				yes	yes	pronounced	no	no	no	25.08	32.43	30.00	33.34
11	PRKAG2	R302Q		34	M	yes/ PM	yes		yes (RF)		yes	yes	pronounced	no	no	no	26.09	24.85	22.12	38.49
12	PRKAG2	R302Q		36	M	yes/ PM	yes*	paroxysmal			yes	yes	pronounced	no	no	no	21.33	39.09	29.40	20.30
13	PRKAG2	R302Q		46	F	yes	yes	paroxysmal	yes (RF)		yes	yes	pronounced	no	no	no	18.24	29.70	24.85	34.85
14	PRKAG2	R302Q		39	M	yes	yes	paroxysmal			yes	yes	pronounced	yes mild	no	no	x	51.22	31.52	10.00
15	PRKAG2	R302Q		30	M	yes/ PM	yes			persistent	yes	yes	pronounced	no	no	no	23.00	32.43	21.52	39.09
16	PRKAG2	H401Q		19	F	yes	yes			paroxysmal	yes	yes	pronounced	no	no	no	21.68	45.76	30.91	15.46
17	PRKAG2	H401Q		18	F	yes	yes				yes	yes	pronounced	no	no	no	21.35	42.12	36.37	10.31
18	PRKAG2	R302Q		22	M	yes	yes				yes	yes	pronounced	no	no	no	21.97	34.25	20.61	33.03
19	transplant		24	64	M	no	no				no	no		no	no	yes mild	13.95	54.58	28.05	13.11
20	transplant		31	53	M	no	no				no	yes	mild	no	no	no	16.28	55.46	25.76	9.70
21	transplant		29	55	M	no	no				no	no		yes mild	no	no	13.03	55.28	32.83	2.78
22	transplant		18	52	M	no	no				no	no		no	no	no	16.51	59.88	28.65	5.95
23	transplant		26	61	F	no	no				no	no		no	no	no	16.42	61.79	29.45	4.24
24	transplant		24	56	M	no	no				no	no		no	no	no	17.49	58.79	31.05	3.99
25	transplant		33	33	F	no	no				no	yes	mild	yes mild	no	no	12.77	x	x	x
26	transplant		30	62	M	no	no				yes	no		no	no	no	18.24	x	x	x
27	transplant		15	45	M	no	no				yes	no		yes mild	no	yes mild	21.15	x	x	x
28	transplant		20	68	F	no	no				yes	no		no	no	no	18.18	58.71	30.73	3.97
29	transplant		47	55	M	no	no				yes	no		no	no	no	21.21	49.70	40.30	2.73

*: indicates that pre-excitation disappeared after atrioventricular block. F: female; M: male; ECG: electrocardiogram; LV: left ventricle; HE: Hematoxylin eosin; PM: pacemaker; RF: radiofrequency ablation.

### Endomyocardial biopsy assessment

#### Histological analysis

Patients with *PRKAG2* cardiomyopathy exhibited marked cardiomyocyte enlargement, pronounced vacuolization, and absence of interstitial fibrosis, except for two cases (patients 8 and 14) with mild fibrosis ( Tables 1 and 2 ). No inflammatory cell infiltration was observed ( Figure 1 ). In contrast, control samples showed mild inflammatory infiltrate in 2 of 11 cases (18.1%), normal cardiomyocyte architecture, mild interstitial fibrosis in 3 cases (27.2%), and cytosolic vacuoles in a minority of myocytes in 2 cases (18.1%) ( Figure 2 ). Transplant recipients whose donors had systemic hypertension exhibited larger cardiomyocytes than those whose donors were normotensive (15.2 ± 1.9 µm vs. 19.7 ± 1.7 µm; p = 0.0037).

**Table 2:** – Histopathological findings of cardiac tissue

	Heart Transplant (n= 11)	PRKAG2 cardiomyopathy (n= 17)	p-value
Fibrosis	2/11 (18%)	2/17 (12%)	0.3697^a^
Inflammation	2/11 (18%)	0/17 (0%)	0.1967^a^
Vacuolization	2/11 (18%)	17/17 (100%)	<0.0001^a^
Glycogen (TEM)	0/8 (0%)	17/17 (100%)	<0.0001^a^
Cardiomyocyte diameter m)^#^	16.84 ± 2.86	21.50 ± 3.23	0.0018^b^

^#^: Data presented as means of 12-100 cardiomyocytes/patient;^a^: Fisher’s exact test;^b^: Student’s unpaired t-test; TEM: Transmission electron microscopy.

**Figure 1:** – Histopathological findings in a patient with PRKAG2 cardiomyopathy. Histological sections stained with Masson’s trichrome (A, C) reveal normal myocardial architecture, normal density of mitochondria, absence of fibrosis or inflammation, and pronounced vacuolization of myocytes. In selected spots, vacuolization was not seen (insert B), in contrast with most areas with intense vacuolization of cardiomyocytes (insert C). Transmission electron micrographs (panels D, G) exhibited large amounts of perinuclear (D) and/or intermyofibrillar glycogen (E, F). Panels F and G show β-particles of glycogen as black dots with 30 nm; *: vacuolization; Gly: glycogen; Lp: lipofuscin; Mf: myofibril; Mit: mitochondria.

**Figure 2:** – Histopathological findings from endomyocardial biopsy of a heart transplant recipient. Sections stained with Masson’s trichrome (A, D) revealed normal myocardial architecture without fibrosis, vacuolization, or inflammation, and normal density of mitochondria. A few patients exhibited inflammatory infiltrates (arrows) (B) and some small areas with vacuolization (C, D). Despite the presence of the latter, transmission electron micrographs do not reveal any glycogen deposits (E, F). *: vacuolization; Gly: glycogen; Mf: myofibril; Mit: mitochondria.

#### Transmission electron microscopy

*PRKAG2* cardiomyocytes displayed extensive glycogen accumulation, predominantly in the perinuclear region, with additional deposits in intermyofibrillar and subsarcolemmal areas. Mitochondria and sarcomeres appeared preserved in most myocytes ( Figure 1D -G). Some cardiomyocytes contained β-glycogen particles interspersed among mitochondria. Lipofuscin granules, corresponding to lysosomal residues, were observed in both *PRKAG2* and control samples. Patients with the *H401Q* mutation exhibited identical histological and ultrastructural findings as those with the *R302Q* variant. Notably, none of the control samples showed glycogen accumulation, even in cases with mild vacuolization ( Figure 2 ).

Quantitative TEM analysis revealed a 6-fold increase in glycogen density (p < 0.0001) in *PRKAG2* patients, ~35% decrease in myofibrillar density (p < 0.0001), and no difference in mitochondrial density between groups ( Figure 3 ). Individual volumetric density (Vv) values showed a clear threshold separating *PRKAG2* from controls. *PRKAG2* cases had at least one TEM image with Vv myofibrils < 30% and Vv glycogen > 22%. Control cases did not meet these thresholds. A positive correlation was observed between cardiomyocyte diameter and glycogen accumulation, while myofibril content showed a negative correlation. Mitochondrial volume density showed no correlation with cardiomyocyte diameter ( Table 3 ).

**Figure 3:** – Volumetric densities (Vv) of myofibrils (A, B), mitochondria (C, D), and glycogen (E, F) from PRKAG2 (white bars/circles) and controls (black bars/dots) patients. Data presented as means ± standard deviation (A, C, E) (Number of patients described above bar, Student’s unpaired t-test) or individual values of each patient (B, D, F) (10 fields per patient); Red line indicates a threshold in the parameters between PRKAG2 and control patients.

**Table 3:** – Pearson correlation between cardiomyocyte diameter and volumetric densities obtained by TEM

	Cardiomyocyte diameter (µm)		Vv myofibrils (%)		Vv mitochondria (%)		Vv glycogen (%)	
	Person r	p-value	Person r	p-value	Person r	p-value	Person r	p-value
Cardiomyocyte diameter (µm)			-0,757	< 0.0001	-0,217	0,309	0,706	< 0.001
Vv myofibrils (%)					0,338	0,099	-0,924	< 0.0001
Vv mitochondria (%)							-0,616	< 0.001
Vv glycogen (%)								

## Missing data

Technical issues during TEM processing prevented a reliable assessment in 1 of 18 *PRKAG2* patients. Light microscopy analysis was not possible for one *PRKAG2* patient. Each patient had two sets of samples: one for light microscopy and one for TEM. In total, 5 of 58 datasets (8.6%) were incomplete ( Table 1 ).

## Discussion

The pathological features of *PRKAG2* cardiomyopathy have been described in a limited number of cases, primarily through EMB, autopsy of sudden death victims, or examination of explanted hearts from transplant recipients. These reports suggest that histopathological findings may vary depending on the clinical context, with more pronounced changes observed in explanted hearts and sudden death cases.

Anatomopathological data from the literature (Supplementary Table 1) indicate that morphological findings have been described in only 22 out of 314 patients (7%) with *PRKAG2* cardiomyopathy. When considering only cases that underwent percutaneous right ventricular EMB for diagnostic purposes, this number is even lower, totaling 14 patients (4.5%). The present study is the first systematic analysis of right ventricular EMB in a large cohort of *PRKAG2* cardiomyopathy patients, assessing its diagnostic utility and the specificity of findings compared to a control group of heart transplant recipients whose donors did not have the disease.

The study’s main findings demonstrated that EMB had a high diagnostic yield in our cohort: all *PRKAG2* -positive patients (18/18, 100%) exhibited the characteristic morphological features, including enlarged cardiomyocytes without architectural disarray, marked vacuolization, and a 6-fold increase in glycogen density (p < 0.0001). In contrast, none of the controls showed glycogen accumulation, and only 2/11 displayed focal vacuolization. These results indicate that EMB reliably distinguished *PRKAG2* cardiomyopathy from controls in this study population.

Pronounced cardiomyocyte vacuolization is a well-documented feature of *PRKAG2* cardiomyopathy,^9 - 14 , 18^as observed in previous studies using H&E or Masson’s trichrome staining, typically associated with PAS-positive granules and large amounts of cytosolic glycogen particles in TEM. Although vacuolization was also observed in two control cases, it was focal and limited to a minority of cardiomyocytes, likely due to fixation or processing artifacts ( Figure 2 ). In contrast, *PRKAG2* patients exhibited widespread vacuolization in most cardiomyocytes.

The prevailing explanation for the presence of vacuoles considers them as negative images, an indirect finding resulting from glycogen washout during slide preparation. However, this assumption does not fully account for the presence of vacuoles in some control group members. In one control case (case 20), TEM clearly showed no accumulation of glycogen in the cytosol ( Table 1 ). It is worth noting that a few normal individuals may exhibit higher volumetric density of glycogen ( Figure 3F ), however, they show no accumulation of glycogen as seen in *PRKAG2* patients ( Figure 1D -G *vs* . Figure 2 E-F).

Cardiac hypertrophy is a hallmark of *PRKAG2* cardiomyopathy. We recently reported the diagnostic utility of transthoracic echocardiography, identifying left ventricular hypertrophy in 25/30 (86%) and right ventricular hypertrophy in 26/30 (90%) carriers of pathogenic *PRKAG2* variants.^27^However, at the cellular level, hypertrophy is typically characterized by an increased number of sarcomeric units within cardiomyocytes. Cardiomyocyte diameter enlargement alone is not diagnostic of hypertrophy, as cellular expansion can result from various factors, including excessive storage of intracellular substances such as glycogen, lipids, and water, which can manifest as ventricular hypertrophy on cardiac imaging.

Recent studies using cardiomyocytes derived from human-induced pluripotent stem cells (hiPSC-CMs)^28 , 29^have shown that *PRKAG2* mutations ( *R302Q* and *N488I* ) induce cell enlargement due to glycogen accumulation, driven by a persistent and inappropriate increase in AMPK activity. Notably, inhibition of AMPK activity using compound C reduced glycogen storage and reversed imaging-detected hypertrophy.^29^The β subunit of AMPK contains a regulatory glycogen-binding domain, allowing wild-type AMPK to function as a glycogen sensor,^30^while immunogold microscopy has shown AMPK subunits co-localized with glycogen particles, with the β1 subunit positioned at the periphery of glycogen rosettes.^31^

In our study, we observed a positive correlation between cardiomyocyte enlargement and intracellular glycogen accumulation, alongside an inverse correlation between myofibril volumetric density and cardiomyocyte diameter. Since volumetric density is a relative measure, we cannot rule out a potential increase in myofibril content in the context of larger cardiomyocytes. Interestingly, a study using a transgenic mouse model with cardiac overexpression of the *N488I PRKAG2* mutant, combined with a knock-in mutation in the muscle form of glycogen synthase ( *GYS1* )–which inhibits glucose-6-phosphatase-stimulated glycogen synthase activity–successfully rescued the glycogen storage phenotype and ventricular pre-excitation. However, it did not affect cardiac hypertrophy or cardiomyocyte enlargement, suggesting that mechanisms beyond glycogen accumulation contribute to hypertrophy in *PRKAG2* cardiomyopathy.^32^

An important finding of our study was that even *PRKAG2* patients without apparent cardiac hypertrophy exhibited morphological changes, including increased glycogen content, absence of inflammation, and minimal or no fibrosis ( Table 1 ). While cardiomyocyte vacuolization occurs in normal hearts and other diseases, it is far more pronounced and widespread in *PRKAG2* cardiomyopathy ( Figure 1 ). Moreover, large glycogen deposits identified on TEM were highly specific to *PRKAG2* and absent in controls. Exceptions include cases with interstitial fibrosis and architectural disarray, reported in rare *PRKAG2* variants such as *K485E* and *E506K* .^3 , 6^Findings from explanted or autopsied hearts in advanced disease also differ, showing more fibrosis,^7 , 8 , 14 , 16 - 19^architectural disarray,^16 , 17^and occasional myocyte degeneration.^14 , 17^These “end-stage” features have also been reported in common *PRKAG2* variants like *R302Q* and *N488I* .^16 , 17^

In this study, genotype-phenotype correlation was consistent within our cohort. All family members of a proband with a *PRKAG2* variant and characteristic clinical features were also carriers of the same variant, in line with previous reports describing the strong association between *PRKAG2* mutations and this phenotype. This complete concordance in our series reinforces the robustness of the genetic-clinical association, while avoiding overgeneralization beyond the studied population.

Other conditions need to be differentiated from *PRKAG2* cardiomyopathy. Hydroxychloroquine-induced cardiotoxicity, typically resulting from long-term use for connective tissue diseases, also presents with pronounced myocyte vacuolization, which extends to skeletal muscle. EMB in this context shows focal interstitial fibrosis without inflammation. Electron microscopy reveals extensive lysosomal inclusions, myeloid bodies, and curvilinear inclusions but no glycogen accumulation.^33^Danon disease ( *LAMP2* mutation), which exhibits a predominant severe cardiac phenotype in young males (usually below 20 years),^34^has a distinct microscopic profile. Post-mortem examination of 2 hearts showed massive hypertrophy, substantial myocyte disarray, and fibrosis, with prominent clusters of vacuolated myocytes and inclusion and inclusions of amorphous granular material in some cells inside scarring regions. Anderson-Fabry disease, which may have a dominant cardiac phenotype, is associated with multisystem involvement.^35^Histologically, it shows extensive cardiomyocyte vacuolation, fibrosis, and myocyte disarray, particularly in the right ventricle, with occasional myocyte necrosis and foamy macrophage replacement. However, no glycogen accumulation is observed. Additionally, mitochondrial cardiomyopathies, such as those associated with the *3243A>G* mutation, can present with an HCM-like phenotype, often misdiagnosed as isolated cardiomyopathy. These patients may lack stroke-like episodes (MELAS syndrome), and TEM typically reveals mitochondrial proliferation.^36^

### Limitations

The control group consisted of cardiac transplant patients whose donor hearts were significantly younger than those with *PRKAG2* cardiomyopathy. However, the pathological changes characteristic of *PRKAG2* cardiomyopathy are not expected to arise from normal aging. Some data were lost because of problems during fixation or sample processing (for TEM: three cases in the control group and one in the *PRKAG2* cardiomyopathy group; for light microscopy: one case in the *PRKAG2* cardiomyopathy group). Given there were only 2/36 missing sets (5.5%) in the *PRKAG2* cardiomyopathy group, it is unlikely that this had a significant impact on our findings.

## Conclusion

Percutaneous right ventricular EMB findings reveal hallmark morphological changes diagnostic of *PRKAG2* cardiomyopathy in all genotypes of positive patients. TEM plays an important role in detecting substantial cardiomyocyte glycogen accumulation. The histopathological and ultrastructural abnormalities described in this study were not observed in control samples. Our findings suggest that EMB, coupled with light and electron microscopy, can aid in diagnosing *PRKAG2* cardiomyopathy. Further studies are needed to validate microscopy as a diagnostic tool for *PRKAG2* cardiomyopathy.

## Supplemental Materials

Supplementary Table 1
